# Hyperglycaemia and Chronic Obstructive Pulmonary Disease

**DOI:** 10.3390/diagnostics13213362

**Published:** 2023-11-01

**Authors:** Mario Cazzola, Paola Rogliani, Josuel Ora, Luigino Calzetta, Davide Lauro, Maria Gabriella Matera

**Affiliations:** 1 Unit of Respiratory Medicine, Department of Experimental Medicine, University of Rome ‘Tor Vergata’, 00133 Rome, Italy; 2 Division of Respiratory Medicine, University Hospital Fondazione Policlinico Tor Vergata, 00133 Rome, Italy; 3 Unit of Respiratory Disease and Lung Function, Department of Medicine and Surgery, University of Parma, 43121 Parma, Italy; 4 Unit of Endocrinology and Metabolic Diseases, Department of Systems Medicine, University of Rome ‘Tor Vergata’, 00173 Rome, Italy; 5 Division of Endocrinology and Diabetes, University Hospital Fondazione Policlinico Tor Vergata, 00133 Rome, Italy; 6 Unit of Pharmacology, Department of Experimental Medicine, University of Campania ‘Luigi Vanvitelli’, 81138 Naples, Italy

**Keywords:** chronic obstructive pulmonary disease, hyperglycaemia, lung function, pharmacological interferences, systemic inflammation, type 2 diabetes mellitus

## Abstract

Chronic obstructive pulmonary disease (COPD) may coexist with type 2 diabetes mellitus (T2DM). Patients with COPD have an increased risk of developing T2DM compared with a control but, on the other side, hyperglycaemia and DM have been associated with reduced predicted levels of lung function. The mechanistic relationships between these two diseases are complicated, multifaceted, and little understood, yet they can impact treatment strategy. The potential risks and benefits for patients with T2DM treated with pulmonary drugs and the potential pulmonary risks and benefits for patients with COPD when taking antidiabetic drugs should always be considered. The interaction between the presence and/or treatment of COPD, risk of infection, presence and/or treatment of T2DM and risk of acute exacerbations of COPD (AECOPDs) can be represented as a vicious circle; however, several strategies may help to break this circle. The most effective approach to simultaneously treating T2DM and COPD is to interfere with the shared inflammatory substrate, thus targeting both lung inflammation (COPD) and vascular inflammation (DM). In any case, it is always crucial to establish glycaemic management since the reduction in lung function found in people with diabetes might decrease the threshold for clinical manifestations of COPD. In this article, we examine possible connections between COPD and T2DM as well as pharmacological strategies that could focus on these connections.

## 1. Introduction

There is increasing evidence that chronic obstructive pulmonary disease (COPD) and type 2 diabetes mellitus (T2DM) often coexist and the presence of T2DM is associated with a worse prognosis [1]. The mechanistic relationships between these two diseases are complex, multifaceted and poorly understood, but may have implications for treatment strategies.

## 2. T2DM in Patients with COPD

A retrospective community-based study in Italy found that COPD patients had a higher incidence of T2DM (18.7%) than the general population (10.5%) [2]. In that study, women with COPD were significantly more likely to develop T2DM than women without COPD [2]. Another population-based study in Taiwan [3] found that T2DM was present in 16% of COPD patients and 19% of COPD patients were newly diagnosed with T2DM over 10 years. The incidence and prevalence of T2DM in COPD patients were also significant in the UK, with a prevalence of 9% or 111,240 cases out of 1.2 million COPD subjects [4].

Non-emphysematous COPD, identified by airflow limitation and absence of emphysema on chest CT scans, is associated with a higher risk of developing DM [5]. A study that calculated the prevalence and incidence of T2DM in COPD patients and investigated the risk factors associated with the development of T2DM reported that the incidence of T2DM in COPD patients was 1.26 per 100 patient-years (95% confidence interval (CI): 1.24–1.28) and was higher in men (1.32 vs. 1.18) [4]. A study of the relationship between COPD and T2DM using the database of an Italian university hospital suggested that T2DM is closely associated with COPD in obese individuals only and affects only those with moderate to severe COPD; it has no effect on those with mild COPD [6].

People with frequent acute exacerbations of COPD (AECOPD) and cardiac comorbidities such as hypertension and heart disease are more likely to develop T2DM [3]. When comparing frequent exacerbators (≥2 treated exacerbations per year) with infrequent exacerbators, the adjusted odds ratio (OR) for T2DM was 1.47 (95% CI: 1.36–1.60); for patients receiving high-dose inhaled corticosteroids (ICS) (>800 mg budesonide equivalent dose), the OR was 1.73 (95% CI: 1.65–1.82) compared to patients not receiving ICS therapy [4]. Regardless of cofactors such as age, gender, history of DM and severity of COPD, each 1 mmol/L (18 mg/dL) increment in blood glucose increases the absolute risk of adverse outcomes in patients with AECOPD by 15% [7]. Diabetic patients hospitalised with AECOPD have higher direct hospital costs due to their more severe profiles [8].

## 3. COPD in Patients with T2DM

On the other hand, people with T2DM have a higher incidence of COPD than controls, with 10% of those with DM also suffering from COPD [9]. The co-occurrence of T2DM and COPD is more common in women than in men and in young than in old people, regardless of gender [10]. There is a non-linear relationship between T2DM duration and COPD risk, but when compared to patients with a T2DM duration of less than one year, those with a T2DM duration of seven years or more had a 46% greater risk of overall mortality [11]. People with T2DM and COPD are more likely to die from all causes than those without COPD [12]. Specifically, they have significantly higher respiratory death rates and moderately elevated lethality for cardiovascular diseases.

Studies have shown that DM is associated with a decline in lung function. This decline in lung function persists after the onset of DM. According to the Framingham Heart Study, lower forced vital capacity (FVC) and adjusted mean residual forced expiratory volume in one second (FEV_1_) are associated with a diagnosis of DM [13]. An association between a new diagnosis of DM and reduced lung function was demonstrated in the Copenhagen City Heart Study [14]. This association was more pronounced in diabetics treated with insulin than in those treated with oral hypoglycaemic agents. Compared with people who do not develop DM, those who are susceptible to the disease have reduced lung function several years before diagnosis [15]. The decline in lung function may begin with prediabetes, according to a recent Chinese study, which showed that both prediabetics and diabetics had worse baseline lung function than the general population [16]. According to the study, FVC, FEV_1_, FVC% and FEV_1_% decreased by 25 mL, 13 mL, 0.71–1.03% and 0.46–0.72%, respectively, with each 1 mmol/l increase in fasting plasma glucose.

Interestingly, diabetic subjects with good glycaemic control (haemoglobin A1c [HbA1c] < 7.0%) show a negative correlation between HbA1c and FEV_1_, whereas this relationship is absent in subjects with poor glycaemic control [17]. In people with T2DM, visit-to-visit variability in fasting plasma glucose and HbA1c is a significant indicator of COPD [18]. In a study conducted in France, patients with DM had significantly lower FVC, FEV_1_, mean expiratory flow, expiratory residual volume, total lung capacity and diffusing capacity for carbon monoxide compared to non-diabetic obese patients [19]. The study also showed that hyperglycaemia leads to mucus overproduction in the airways [20]. Elevated blood glucose levels, with or without DM, are strongly associated with an increased risk of subsequent severe AECOPD. In a Chinese study, high and moderate HbA1c levels were significantly associated with a higher risk of severe AECOPD (hazard ratio [HR], 2.74, 95% CI: 1.70–4.41, *p* < 0.01, and HR, 2.19, 95% CI: 1.39–3.46, *p* < 0.01, respectively) [21]. This association persisted even after controlling for potential confounders, including age, sex, body mass index, smoking status, duration of COPD, frequency of hospitalisation for AECOPD in the previous year, GOLD stages, COPD Assessment Test (CAT) score, use of corticosteroids, hypertension and cardiovascular disease. Subgroup analysis revealed a significant association between HbA1c levels and the likelihood of developing severe AECOPD in different GOLD stages and DM. Systematic analysis suggests that hyperglycaemia increases susceptibility to bacterial infection and thus increases the prevalence of severe AECOPD [22]. Patients with T2DM appear to have a higher risk of hospitalisation during AECOPD [23].

## 4. Potential Mechanisms of the Links between COPD and T2DM

It is crucial to ascertain whether COPD precedes T2DM or vice versa, or whether both conditions develop simultaneously via a single pathogenic substrate [24].

The growing incidence of T2DM in COPD has been attributed to several mechanisms including increased cigarette smoking, corticosteroid exposure, disease-related inflammation, oxidative stress, hypoxia, rising obesity and declining physical activity (Table 1) [25]. Studies have found a correlation between obesity and COPD. However, there appears to be a discrepancy among overweight patients. Whilst obesity is often associated with less severe airflow obstruction, it is also linked to increased symptoms of dyspnoea, decreased health-related quality of life and greater reliance on inhaled medications [26,27]. Additionally, there seems to be a protective effect on mortality, although its impact varies depending on the individual’s body mass index (BMI). A study that utilised information from the National Health and Nutrition Examination Survey (NHANES III) found that individuals with a BMI ≥ 40 kg/m^2^ faced an increased risk of death from respiratory illness [28]. A cohort study discovered that cigarette smoke contributed to 30% of cases in T2DM, as opposed to 26% of cases in non-diabetics when the attributable risk of COPD was established [29]. Individuals who smoke are twice as likely to develop T2DM compared to those who do not smoke. This correlation may be due to the fact that smoking leads to oxidative stress, which exacerbates insulin resistance and alters energy production [30]. A large retrospective nested case-control study conducted in Britain revealed a decrease in COPD occurrence among individuals with T2DM when compared to matched controls. This is likely due to beneficial changes in lifestyle, particularly successful smoking cessation [31]. However, quitting smoking can also result in an increased probability of developing T2DM in the near future, with such a rise largely being caused by weight gain and systemic inflammation [32]. The elevated incidence of concomitant chronic diseases, including T2DM and COPD, could stem from systemic inflammation caused by activation of the innate immune system [24].

Smoking-related inflammation has apparent consequences in COPD [28] but it is possible that another condition, such as a concomitant disease, also plays a part in the systemic inflammation that ultimately affects the lungs [33]. It is intriguing to note that independent of smoking behaviour, a robust effect-size connection exists between BMI and COPD [34]. Because of excessive adipose tissue lipolysis, high levels of circulating fatty acids, and consequent incorrect activation of the inflammatory cascade, obesity can be seen as an inflammatory condition [35]. Tumour necrosis factor-α (TNF-α) and interleukin-6 (IL-6) are examples of proinflammatory cytokines that are known to cause insulin resistance by inhibiting insulin receptor signalling and hence raise the risk of T2DM [33]. There is experimental evidence that lung inflammation somewhat inhibits insulin action in peripheral organs and attenuates insulin-induced inhibition of hepatic glucose synthesis [36]. FEV_1_ and FVC are adversely correlated with higher levels of insulin resistance [37].

Oxidative stress from both environmental and cellular sources is a significant component causing and/or exacerbating COPD [38]. It can come from both cellular and environmental sources and develops because of endogenous antioxidant defences being overpowered by reactive oxygen species (ROS), which may be linked to increased inflammation [35]. Oxidative stress causes insulin insensitivity by inducing a variety of stress pathways, mainly the advanced glycation end product (AGE)-receptor for AGR (RAGE) pathway, which finally stimulates further mitochondrial impairment, and the protein kinase C (PKC) route with its large family of serine/threonine kinases. These are thought to be the two main pathways leading to excessive ROS generation caused by high hyperglycaemia, insulin resistance and, consequently, hyperglycaemia reinforcement [39,40]. Conversely, hyperglycaemia induces mitochondrial damage, collateral glucose routes and spontaneous glucose reactions [40]. Oxidative stress is a result of all processes that encourage the production of excessive ROS. It has been suggested that a decrease in endogenous antioxidants because of lower levels of the transcription factor nuclear factor-erythroid 2 related factor 2 (Nrf2), which regulates many antioxidant genes in patients with both COPD and T2DM, could be another plausible mechanism leading to systemic oxidative stress, which may be a fundamental amplifying mechanism in both diseases [39].

There is also evidence that hypoxia, which is frequent in patients with advanced COPD, leads to decreased insulin sensitivity, increased lipolysis, and poor glucose tolerance [24].

Patients with T2DM have been shown to have accelerated deterioration of lung function [41]. High glucose concentrations potentiate the Rho-associated coiled-coil-containing protein kinase (ROCK) pathway, which leads to intracellular calcium mobilization and phosphorylation of myosin-targeting subunit-1 (MYPT-1) [42]. It implies accentuated intracellular calcium release in cultured human airway smooth muscle (ASM) cells and increased response of ASM to contractile agents. This finding may play a crucial role in the reduced lung function observed in patients with DM. However, at least in guinea pigs, insulin also causes ASM contraction. This is probably because it produces contractile prostaglandins, which rely on Rho-kinase to exert a contractile impact [43]. The lung has insulin receptors, and high insulin binding concentrations may exacerbate ASM constriction and hyperresponsiveness [44].

Chronic hyperglycaemia also induces the formation of AGEs through non-specific glycation. AGEs cause serious complications, such as the disruption of endothelial cells with subsequent damage to the macro- and microvasculature [45]. Microvascular and parenchymal changes in the lung cause systemic hypoxia and disrupt energy metabolism. When AGEs attach to RAGE, they also have significant signalling action. Through persistent stimulation of the nuclear factor-kB (NF-kB) pathway, the AGE–RAGE combination stimulates the production of inflammatory genes in target cells [46,47]. NF-kB activation in the lung reduces hepatic glucose synthesis by insulin resulting in insulin resistance in peripheral tissues [36]. However, it has been proposed that inflammation in the lung airway epithelium alters glucose homeostasis by reducing the number of skeletal muscle capillaries available to transfer glucose and insulin to myocytes without necessarily affecting insulin signalling [48]. In addition, AGEs also cause T2DM patients’ mononuclear cells to secrete higher plasma CRP and TNF [49]. Patients with COPD have overexpressed RAGE in their ASM and airway epithelium [50].

## 5. COPD and T2DM: Mutual Pharmacological Interferences

It is important to always consider the potential risks and benefits for patients with T2DM treated with pulmonary medications (Table 2) and for patients with COPD taking antidiabetic medications (Table 3) [51]. The relationship between COPD therapy, infection risk, T2DM and AECOPD risk has been described as a vicious circle; however, several interventions may be able to break this cycle [52].

## 6. Potential Impact on T2DM of Drugs Used to Treat COPD

The use of COPD medications is generally safe in patients with T2DM, but the effect of ICS on DM remains uncertain [53]. A systematic review and meta-analysis of 17 RCTs involving 43,430 subjects concluded that in patients with COPD, the use of ICS does not affect blood glucose levels and is not associated with the incidence of new-onset DM or its progression [54]. However, a small association between ICS use and the presence of T2DM with the occurrence of hyperglycaemia in susceptible individuals has been reported in the literature [6]. Furthermore, an increased risk of DM, DM progression and osteoporosis was observed in diabetic patients treated with long-term ICS therapy for COPD with a mean daily exposure of ≥ 500 µg fluticasone propionate equivalent [55]. A historical matched cohort study using primary care medical record data from two large UK databases showed that in people with COPD and comorbid T2DM, cumulative ICS dose exposure of >250 µg (fluticasone equivalents) therapy may make DM control more difficult, with significantly greater increases in HbA1c levels, more annual DM-related general practice visits and more frequent prescriptions for glucose strips, compared with those not treated with ICS [56].

Blood glucose control is also affected by inhaled β_2_-agonists. Their acute administration is ‘pro-diabetogenic’ due to rapid increases in plasma glucose and insulin concentrations [57]. β_2_-agonists can affect glucose homeostasis by regulating insulin and glucagon secretion, hepatic glucose production and muscle glucose uptake, resulting in increased blood glucose levels [58]. Acute administration of inhaled formoterol and salmeterol has been shown to cause statistically significant dose-related increases in plasma glucose [59]. However, this effect diminishes over time and longer-term β_2_-agonist therapy is not associated with higher plasma glucose and insulin levels [60]. Furthermore, these effects appear to be of little clinical significance unless the patient is close to developing glucose intolerance [61]. In fact, the average increase caused by the maximum dose of inhaled formoterol was small (2.6 mmol/L). However, this may be sufficient to meet current standards for impaired fasting glucose or even DM (126 mg/dL or 7 mmol/L) [62]. In any case, because of the risk of ketoacidosis, β_2_-agonists should be used cautiously in diabetics [58]. In patients with COPD who are also receiving corticosteroids, resulting in weight gain, inactivity and insulin resistance, the additional effects of β_2_-agonists on glycaemic dysregulation may be difficult to detect but substantial [61]. Nevertheless, the combination of an ICS and a β_2_-agonist has been shown to reduce the likelihood of a COPD patient developing T2DM [6]. Interestingly, β_2_-agonists have been shown to protect against the vascular effects of DM, an effect that is likely to be linked to the β-arrestin2/inhibitor of NF-kB (IkB)α/NF-kB pathway, resulting in a reduction in inflammatory stimuli and tissue protection [63].

In a pooled safety study of tiotropium, there was no higher overall risk of DM in those who received this bronchodilator than in those who received a placebo [64]. However, the relative risk (RR) of hyperglycaemic episodes was 1.69. In addition, tiotropium was associated with DM (OR = 1.6; 95% CI: 1.0–2.5; *p* = 0.034) in a Spanish study conducted to determine its comorbidities and economic impact on COPD [65]. Paracrine release of acetylcholine (ACh) from pancreatic alpha-cells has been shown to directly trigger beta-cell insulin production via M_3_ and M_5_ muscarinic receptors [66]. Similarly, cholinergic paracrine output has been shown to stimulate delta cells to release somatostatin via the M_1_ receptor. Somatostatin, on the other hand, greatly reduces insulin secretion. This indicates that selective blockade of pancreatic M_3_ or M_5_ receptors may affect insulin production and contribute to the development of T2DM [67]. Indeed, there is evidence that oral antimuscarinic drugs may impair late-phase insulin activity to varying degrees in diabetic patients [68].

Some molecular evidence suggests that alpha-1-antitrypsin (AAT) may be involved in T2DM; in mice, obesity and insulin resistance are caused by an imbalance between AAT and neutrophil elastase [69]. AAT regulates fatty acid oxidation, energy expenditure and AMP-activated protein kinase processes. AAT-targeted therapies are playing an increasing role in the treatment of T1DM [70]. They may be useful in treating existing T1DM but also in slowing the progression of newly diagnosed T1DM. The importance of AAT in the pathophysiology of T2DM is less clear and further studies are needed to understand this association and potential treatment strategies [70].

## 7. Potential Impact on COPD of Drugs Used to Treat T2DM

According to a Taiwanese cohort study, people with T2DM and COPD who require insulin therapy may be more likely to develop AECOPD, pneumonia, need to use a ventilator, and have severe hypoglycaemia; although they are not significantly more likely to die from the disease [71]. There is also evidence that insulin therapy plays a role in the development of lung cancer [72]. The proliferation of primary human ASM cells, which exhibit a marked increase in calcium sensitivity and mitochondrial respiration upon insulin exposure, is significantly promoted by insulin treatment (1 μg/mL) and collagen release is also stimulated [73]. In addition, insulin increases β-catenin signalling, which is involved in ASM cell contraction [74], by triggering the phosphatidylinositol 3′-kinase (PI3K)/protein kinase B (Akt) signalling pathway and inhibiting glycogen synthase kinase-3β through phosphorylation [73]. Interestingly, research indicates that hyperinsulinemia enhances vagally produced bronchoconstriction by limiting neuronal M_2_ muscarinic receptor activity and increasing ACh release from airway parasympathetic neurons [75].

As previously mentioned, high glucose concentrations increase the ASM response to contractile agents [42]. Therefore, maintaining blood glucose levels in the normal range is critical as the decline in lung function observed in diabetic patients may lower the threshold for clinical signs of COPD [24]. As the low-grade systemic inflammatory pathway appears to be the common link between COPD and T2DM [24], it is appropriate to explore a treatment approach that lowers blood glucose and has an anti-inflammatory effect when treating these two diseases simultaneously [51].

Oral hypoglycaemic agents, mainly biguanides, thiazolidinediones and dipeptidyl peptidase-4 (DPP-4) inhibitors, are now known for their anti-inflammatory properties, whereas other oral hypoglycaemic agents, such as sulfonylureas, meglitinides, and α-glucosidase inhibitors, have not been thoroughly studied for their effects on inflammation and COPD [76]. 

However, an analysis of data from Taiwan’s National Health Insurance Research Database showed that sulfonylureas reduce the risk of cardiovascular and bacterial pneumonia events, mortality and ventilation use, in patients with COPD and T2DM [77]. It has also been documented that 91–180 days of sulfonylureas therapy mitigated the risk of severe AECOPD by 28% (adjusted OR, 0.72; 95% CI: 0.58–0.90), while longer treatments consistently produced 24–30% lower risks [76].

Conversely, another Taiwanese nationwide cohort study found that metformin use in patients with T2DM and COPD was associated with a higher risk of bacterial pneumonia, hospitalisation for COPD and use of invasive mechanical ventilation [78]. Furthermore, this biguanide had no detectable effect on C-reactive protein or clinical outcomes in patients hospitalised for AECOPD in a study that was too short (1 month) to demonstrate the true impact of metformin on systemic inflammation [79]. However, among patients with coexisting COPD and T2DM, metformin users from Taiwan had a significantly lower risk of death over 2 years (HR, 0.46; 95% CI: 0.23–0.92) than non-metformin users [80]. There was no difference in survival between metformin users and COPD patients without T2DM. In addition, metformin was associated with a reduced rate of death from chronic lower respiratory disease in people with DM in a cohort representative of the US population [81]. Metformin may help patients with COPD and T2DM by reducing hospitalisations, mortality and symptoms, according to a comprehensive analysis that included six publications and 3467 people [82]. It was also shown that metformin use was associated with less emphysema progression over time [83] and a numerical increase in FEV_1_, but also significantly improved FVC in the T2DM population without coexisting COPD [84]. However, when patients were also treated with insulin, no changes in lung function were found, probably because of a possible antagonistic interaction between the pleiotropic effects of metformin, leading to relaxation of the ASM, and the increased contractile ASM tone induced by insulin [84].

Although thiazolidinedione use was associated with a higher risk of cardiovascular events, mechanical ventilation, pneumonia, and lung cancer in patients with T2DM and COPD [85], further analysis of the Taiwan National Health Insurance database documented that pioglitazone use was linked with a significantly lower risk of COPD, especially when used for more than 11 months [86]. The overall HR for COPD compared with never users was 0.778 (95% CI: 0.667–0.908). No interactions were found between pioglitazone and COPD risk factors, including pneumonia, pulmonary tuberculosis, and tobacco abuse. Furthermore, data from US veterans with DM and COPD showed that exposure to thiazolidinediones was associated with a small but significant reduction in the risk of AECOPD [87]. In addition, combination therapy with thiazolidinediones was associated with a reduced incidence of AECOPD in patients with advanced T2DM and concomitant COPD, according to a cohort-based case-control study using data from Taiwan’s National Health Insurance Research Database [88]. Patients receiving sulfonylurea and thiazolidinedione had a lower risk of AECOPD than those receiving metformin and sulfonylurea, and those receiving metformin, sulfonylurea and thiazolidinedione had a lower risk of AECOPD than those receiving a combination of metformin, sulfonylurea, and α-glucosidase inhibitor. This trend was independent of the complexity of the patient’s COPD.

A meta-analysis of randomised trials of sodium–glucose transporter 2 (SGLT2) inhibitors showed a trend towards a reduced risk of COPD with these drugs (RR, 0.79, 95% CI: 0.61–1.02) [89]. Another recent meta-analysis confirmed that SGLT2 inhibitors were associated with a reduced risk of COPD compared with placebo (RR 0.77; 95% CI: 0.62–0.96), regardless of whether they were used at high or low doses [90]. However, dapagliflozin did not induce better glycaemic control than placebo in patients with prednisone-induced hyperglycaemia during AECOPD [91]. In any case, by linking data from the Gp OnLine Data and Aurum databases of the Clinical Practice Research Datalink with the Hospital Episode Statistics Admitted Patient Care and Office for National Statistics databases, it was observed that SGLT-2 inhibitors were associated with a 38% lower risk of severe exacerbations compared with sulfonylureas (2.4 vs. 3.9 events per 100 person/years; HR, 0.62; 95% CI: 0.48–0.81) but not moderate exacerbations (HR, 1.02; 95% CI: 0.83–1.27) [92]. In addition, the use of SGLT2 inhibitors is associated with a lower risk of obstructive airway disease and a lower rate of exacerbations of obstructive airway disease in clinical practice compared with the use of DPP4 inhibitors [93]. This is not unexpected, as DPP-4 inhibitors were associated with a slightly lower incidence of severe exacerbations (4.6 vs. 5.1 events per 100 person-years; HR, 0.91, 95% CI 0.82–1.02) and moderate exacerbations (HR, 0.93; 95% CI: 0.82–1.07) compared with sulfonylureas [92]. However, in an experimental model of chronic asthma, sitagliptin demonstrated its ability to reduce inflammation and airway remodelling [94] while alogliptin improved lung function in obese subjects with T2DM [95].

Another option is the use of an oral antidiabetic with direct broncholytic activity [24]. Exendin-4 is a glucagon-like peptide (GLP)-1 agonist that activates the GLP-1 receptor (GLP-1R) and relaxes isolated human bronchi via the cAMP-protein kinase A pathway [96]. GLP-1 has also been shown to restore dysregulated arginine metabolism and reduce AGE-induced inflammation [97]. Regardless of blood glucose levels, GLP-1R agonist therapy improves airway function in people with T2DM who do not have underlying COPD [84].

Another GLP-1 agonist, liraglutide, also increased FVC in patients with T2DM [98]. Compared with sulfonylureas, GLP-1R agonists were reported to be associated with a 30% lower risk of severe AECOPD (3.5 vs. 5.0 events per 100 person-years; HR 0.70; 95% CI: 0.49–0.99) and moderate AECOPD (HR 0.63; 95% CI: 0.43–0.94) in patients with T2DM and COPD [92]. 

Recent retrospective research has confirmed this finding: the use of GLP-1R agonists was associated with a significantly lower risk of moderate exacerbations compared with sulfonylurea users and a significantly lower risk of severe exacerbations compared with DPP-4 inhibitor and sulfonylurea users [99]. GLP-1R agonists were associated with a significantly lower incidence of severe exacerbations than DPP-4 inhibitors and sulfonylureas, and moderate exacerbations than sulfonylureas. After correcting for clinical variables, those using GLP-1R agonists had a reduced incidence of moderate exacerbations compared with those using DPP-4 inhibitors. There was no significant difference in exacerbation outcomes between GLP-1R agonists and SGLT2 inhibitors. GLP-1R agonists may significantly improve clinical symptoms in COPD patients, alleviate airflow limitation, influence airway damage, fibrosis and remodelling, shorten hospital stays, reduce patients’ financial burden, reduce the risk of COPD-related complications, improve patients’ long-term prognosis, and may be a novel target for the prevention and treatment of COPD [100].

## 8. Conclusions

COPD may coexist with T2DM. As already mentioned, patients with COPD have an increased risk of developing T2DM compared with a control. However, hyperglycaemia and DM have been associated with reduced predicted levels of pulmonary function [24]. Therefore, using shared therapeutic strategies for both conditions could be a practical approach. The optimal method for managing T2DM and COPD concurrently is to target the common inflammatory substrate, thus addressing both pulmonary inflammation (COPD) and vascular inflammation (DM). In any case, as impaired lung function in people with DM may lower the threshold for clinical manifestations of COPD, it is essential to implement glycaemic control.

## Figures and Tables

**Table 1:** Potential mechanisms of the links between COPD and T2DM.

Obesity that is associated with increased adipose and systemic inflammation and reduced insulin sensitivity. Cigarette smoke that directly impairs insulin action and reduces peripheral glucose uptake. Oxidative stress from both environmental and cellular sources that promotes insulin resistance and alters energy production. Decrease in endogenous antioxidants. Lung inflammation that induces insulin resistance by blocking signalling through the insulin receptor of proinflammatory cytokines. Chronic low-grade inflammation. Activation of the innate immune system. Impaired adaptive responses to hypoxia due to insufficient activation of hypoxia-inducible factors signalling. Low lung function values that have a higher risk of developing insulin resistance. High glucose concentrations that increase the responsiveness of ASM to contractile agents. Microangiopathy of lung vasculature. Glucotoxicity that activates nonenzymatic glycosylation of lung collagen and elastin by AGEs resulting in reduced lung elasticity. Insulin that induces ASM contraction.

AGEs, advanced glycosylation endproducts; ASM, airway smooth muscle.

**Table 2:** Potential risks and benefits on T2DM of drugs used to treat COPD.

Risks
Increased risk of diabetes, diabetes progression and osteoporosis was observed in diabetic subjects when they were treated with long-term ICS therapy for COPD at a mean daily exposure of ≥500 µg fluticasone propionate equivalent. Acute administration of β_2_-agonists induces rapid plasma glucose and insulin concentration increases. These effects appear to decline over time. At least oral antimuscarinic agents may reduce late-phase insulin action to various degrees of diabetic state.
**Benefits**
Combining an ICS with a β_2_-agonist can reduce the odds of developing T2DM in COPD patients. β_2_-agonists protect against the vascular effects of diabetes, which results in a decrease in inflammatory stimuli and tissue protection. Augmentation therapy with AAT may favourably impact DM.

AAT, alpha-1 antitrypsin; ICS, inhaled corticosteroid.

**Table 3:** Potential risks and benefits of drugs used to treat T2DM on COPD.

Risks
Insulin shows potentiation of ASM contraction. Hyperinsulinemia potentiates vagally induced bronchoconstriction. Insulin therapy may have an increased risk of AECOPDs, pneumonia, ventilator use and severe hypoglycaemia without significantly increasing the risk of death. Insulin therapy plays a role in lung cancer development. Metformin use associated with higher risks of bacterial pneumonia, hospitalisation for COPD and use of invasive mechanical ventilation. Thiazolidinedione use associated with higher risks of cardiovascular events, ventilation use, pneumonia, and lung cancer. Dapagliflozin did not result in better glycaemic control than placebo in participants with prednisone-induced hyperglycaemia during AECOPD.
**Benefits**
Sulfonylureas associated with significantly lower risks of AECOPD, cardiovascular events, ventilation use, bacterial pneumonia and mortality. Metformin may improve health status and symptoms, hospitalisations and mortality. Metformin use associated with lesser emphysema progression over time and with a numerical increase in FEV_1_ and significant improvement in FVC in T2DM population without coexisting COPD. Exposure to thiazolidinediones associated with a small but significant reduction in risk for AECOPD. SGLT2 inhibitors associated with decreased risks of COPD regardless of whether they are used in high or low doses. In patients with T2DM and no underlying COPD, treatment with GLP-1R agonists improves airway function regardless of blood glucose levels. GLP-1R agonist use associated with a significantly reduced risk of severe exacerbations compared to DPP-4.

AECOPD, acute exacerbation of COPD; DPP-4, dipeptidyl peptidase-4; FEV_1_, forced expiratory volume in one second; FVC, forced vital capacity; GLP-1, glucagon-like peptide-1; SGLT2, sodium–glucose transporter 2.

## Data Availability

Not applicable.
